# Development of a screening tool predicting the transition from acute to chronic low back pain for patients in a GP setting: Protocol of a multinational prospective cohort study

**DOI:** 10.1186/1471-2474-9-167

**Published:** 2008-12-19

**Authors:** Markus Melloh, Nikolaus Aebli, Achim Elfering, Christoph Röder, Thomas Zweig, Thomas Barz, Peter Herbison, Paul Hendrick, Suraj Bajracharya, Kirsten Stout, Jean-Claude Theis

**Affiliations:** 1 Section of Orthopaedic Surgery, Department of Medical and Surgical Sciences, Dunedin School of Medicine, University of Otago, Private Bag 1921, Dunedin 9054, New Zealand; 2 Department of Surgery and Orthopaedics, Swiss Paraplegic Centre, Guido A. Zäch Strasse 1, 6207 Nottwil, Switzerland; 3 Department of Work and Organizational Psychology, Institute for Psychology, University of Berne, Muesmattstrasse 45, 3000 Berne 9, Switzerland; 4 MEM Research Center for Orthopaedic Surgery, University of Berne, Stauffacherstrasse 78, 3014 Berne, Switzerland; 5 Department of Orthopaedic Surgery, Asklepios Klinikum Uckermark, Auguststrasse 23, 16303 Schwedt/Oder, Germany; 6 Department of Preventive and Social Medicine, Dunedin School of Medicine, University of Otago, PO Box 913, Dunedin 9054, New Zealand; 7 School of Physiotherapy, University of Otago, PO Box 56, Dunedin 9054, New Zealand

## Abstract

**Background:**

Low back pain (LBP) is by far the most prevalent and costly musculoskeletal problem in our society today. Following the recommendations of the Multinational Musculoskeletal Inception Cohort Study (MMICS) Statement, our study aims to define outcome assessment tools for patients with acute LBP and the time point at which chronic LBP becomes manifest and to identify patient characteristics which increase the risk of chronicity.

**Methods:**

Patients with acute LBP will be recruited from clinics of general practitioners (GPs) in New Zealand (NZ) and Switzerland (CH). They will be assessed by postal survey at baseline and at 3, 6, 12 weeks and 6 months follow-up. Primary outcome will be disability as measured by the Oswestry Disability Index (ODI); key secondary endpoints will be general health as measured by the acute SF-12 and pain as measured on the Visual Analogue Scale (VAS). A subgroup analysis of different assessment instruments and baseline characteristics will be performed using multiple linear regression models.

This study aims to examine

1. Which biomedical, psychological, social, and occupational outcome assessment tools are identifiers for the transition from acute to chronic LBP and at which time point this transition becomes manifest

2. Which psychosocial and occupational baseline characteristics like work status and period of work absenteeism influence the course from acute to chronic LBP

3. Differences in outcome assessment tools and baseline characteristics of patients in NZ compared with CH.

**Discussion:**

This study will develop a screening tool for patients with acute LBP to be used in GP clinics to access the risk of developing chronic LBP. In addition, biomedical, psychological, social, and occupational patient characteristics which influence the course from acute to chronic LBP will be identified. Furthermore, an appropriate time point for follow-ups will be given to detect this transition. The generalizability of our findings will be enhanced by the international perspective of this study.

**Trial registration:**

[Clinical Trial Registration Number, ACTRN12608000520336]

## Background

Low back pain (LBP) is the most prevalent and costly musculoskeletal disease, potentially leading to permanent disability [1,2]. For economically advanced societies a lifetime prevalence of LBP of up to 80%, an annual prevalence of up to 60%, and a point prevalence of up to 40% is estimated [3]. The natural course of LBP is self-limiting whereas an estimated 10% of patients develop chronic LBP as defined by duration of more than 12 weeks [4,5]. Though chronic LBP affects only a small group of patients, it is a high socioeconomic burden exceeding the treatment of acute LBP significantly [6]. About 30% of these costs are direct costs caused by medical treatment, whereas 70% are attributable to indirect costs e.g. loss of production [2].

Hence, it is of high importance to detect patients at risk of developing chronic LBP at an early stage as well as to identify modifiable risk and protective factors [7]. Evidence and outcome-based research is needed to provide information on the appropriate assessment of patients with acute LBP. To identify these patients, prognostic factors of chronic LBP must be known. According to the biopsychosocial model the influence of various factors in addition to severity of LBP has to be taken into account [8,9].

Guidelines already exist for the management of acute LBP [10], but some of the main questions in acute LBP – which assessment tools are the best identifiers for patients at risk for the transition from acute to chronic LBP and at which time point this transition takes place – are not clearly answered. Chronic LBP is defined as LBP lasting longer than 12 weeks [11]. Furthermore, there are inconsistent findings on cross-country differences in commonly used patient outcome assessment tools and baseline characteristics [12-15].

Although screening instruments are not routinely used in medical practice they can help to detect prognostic factors in the course of LBP. They should consist of internationally accepted core outcome measures e.g. as recommended by the MMICS Statement and should be validated on a multinational level [16]. A recent review on screening instruments for the identification of prognostic factors for chronicity in patients with LBP reported that psychological and occupational factors have the highest reliability in predicting an unfavourable outcome [17]. Therefore, in this study protocol we focus on these factors in the design of a new comprehensive screening instrument.

## Methods

### Research plan

#### Objectives

This study aims to identify outcome assessment tools of patients with acute LBP which includes defining patient characteristics at baseline to allow the identification of patients at risk of developing chronic LBP [7,18-28] and at which time the chronicity becomes manifest [29-33]. This study will be undertaken in cohorts of patients in both New Zealand (NZ) and Switzerland (CH) to establish any similarities and differences between them [12-15].

#### Hypotheses

Three hypotheses will be examined:

1. Specific biomedical, psychological, social, and occupational outcome assessment tools are identifiers for the transition from acute to chronic LBP at a time point between 3 and 12 weeks.

2. Specific psychosocial and occupational baseline characteristics like work status and period of work absenteeism influence the course from acute to chronic LBP.

3. There are differences in patient outcome assessment tools and baseline characteristics between patients in NZ and CH, e.g. in disability compensation systems.

#### Study design

In this two arm cohort study, patients will be recruited from GP clinics in NZ and CH. Participants will be assessed directly after attending their GP for LBP treatment for the first time or for recurrent LBP after a pain free period of at least 6 months (T0) with follow-up at 3, 6, and 12 weeks, and at 6 months (T1-4). 6 months are the cut-off point in follow-up as longer follow-ups may incorporate additional errors affecting the identification of predictors.

The study protocol has been approved by the Lower South Regional Ethics Committee (LRS/08/03/008).

#### Inclusion and exclusion criteria

We defined broad inclusion criteria to ensure that the spectrum of patients represents the spectrum seen in routine settings: consecutive patients attending a GP clinic for acute LBP in Otago and Waikato districts, NZ and German speaking CH for a first time or after a pain free period of at least 6 months; a minimum age of 18 years at the time visiting a GP; a maximum age of 65 years; a good understanding of English or German; and a signed consent, are required.

Exclusion criteria are: chronic LBP (defined as LBP continuing for more than 12 weeks at time of first visit to GP) [11,30]; specific LBP (infection, tumour, osteoporosis, ankylosing spondylitis, fracture, deformity, inflammatory process, cauda equina syndrome) [10]; comorbidity that determines overall well-being (e.g. painful disabling arthritic hip joints); pregnancy; expected loss to follow-up (e.g. due to moving from the district); and unwillingness to complete questionnaires (Table 1).

**Table 1:** Inclusion and exclusion criteria

**Inclusion criteria**	**Exclusion criteria**
1. Patients attending GP clinic with acute LBP	1. Chronic LBP
2. Age 18–65 years	2. Specific LBP
3. Good understanding of English (NZ) or German (CH)	3. Comorbidity determining overall well-being
4. Written consent	4. Pregnancy
	5. Expected loss to follow-up
	6. Unwillingness to complete questionnaires

### Outcome assessment

#### Demographic and baseline characteristics

The following demographic, biomedical, psychological, social, and occupational factors will be assessed at baseline according to the recommendations of the MMICS Statement:

- Gender [34,35]

- Age [32,36-38]

- BMI

- Ethnicity

- ODI [39,40]

- Acute SF-12 [41]

- Quality and intensity of LBP (SF-McGill including VAS) [42-44]

- LBP history (duration and recurrence) [45-48]

- Radiating leg pain below the knee

- Lifestyle factors (exercise, smoking, alcohol consumption) [49-52]

- Depression (modified self-rating depression scale by Zung ZUNG) [53,54]

- Somatisation (Modified Somatic Perceptions Questionnaire MSPQ) [55]

- Fear avoidance beliefs (Fear Avoidance Beliefs Questionnaire FABQ) [56-58]

- Pain catastrophizing (Pain Catastrophizing Scale PCS) [59]

- Marital/relationship status

- Social support

- Educational status

- Employment/work status

- Job satisfaction [35,60-62]

- Job control [35,63]

- Work stress factors [61,62,64-67]

- Belief that work has caused LBP [68-70]

- Duration of work-absenteeism [9,71-73]

- Reasons for not working

- Disability compensation status [74-79]

- Patients' expectations on return to work [80-82].

#### Outcome measures

ODI, acute SF-12, and VAS pain score are among the most frequently used outcome assessment measurements of LBP. All of them are valid and reliable. In this study of outcome predictors, the primary endpoint for chronicity is ODI. Key secondary endpoints are acute SF-12 and VAS pain score.

Other secondary outcomes that will be collected by patient self-assessment as recommended by the MMICS Statement are: reduction in normal activities due to LBP; health care utilization including treatment received and referral as provided by the Accident Compensation Corporation (ACC) and GPs; medication [83]; modified self-rating depression scale by Zung (ZUNG); Modified Somatic Perceptions Questionnaire MSPQ (MSPQ); Fear Avoidance Beliefs Questionnaire (FABQ); Pain Catastrophizing Scale (PCS); patient satisfaction with condition and care; return to work (RTW); and sick leave over the last week.

### Data management

#### Database

Patient data will be stored at a central database at the University of Otago (UoO), Dunedin, in strictly anonymised fashion. Data will be analysed in cooperation with a statistician in the Department of Preventive and Social Medicine, Dunedin School of Medicine. Data will be double entered and cleaned before use.

### Statistical analysis

#### Multivariate identification of predictors for chronicity

Changes in primary and secondary endpoints between baseline data and different time points of follow-up will be analysed by multiple linear regression models. Patients with chronic LBP at 6 months follow-up will be compared with patients without chronic LBP at the same time point.

Patients with chronic LBP in this study are defined as patients with an ODI score without improvement by a Minimal Clinically Important Difference (MCID) of 10 ODI points between T0 (attending the GP for the first time or after a pain free period of at least 6 months) and T4 (6 months follow-up) [84-87].

An analysis of different assessment instruments and baseline characteristics will be performed to find appropriate scores or patient characteristics to identify patients at risk to develop chronic LBP. Different patient assessment tools will be allocated to the following four groups: demographic-biomedical, psychological, social, and occupational risk group [88]. We aim to detect at least one assessment instrument in each group which identifies patients at risk.

#### Identification of time of chronicity

In a second step, a subgroup analysis on patients without chronic LBP will be performed to identify the time of follow-up (TX) where this patient group shows an improvement in ODI by at least the MCID. As patients with chronic LBP by definition of their condition don't show an improvement at TX, this follow-up is the most appropriate time point for detecting the transition from acute to chronic LBP in patients at risk.

### Sample size/power calculation

#### Sample size considerations

Sample size considerations in clinical studies start from what is considered a MCID. In studies of chronic LBP, the ODI is commonly used as the main outcome measure, and a difference in 10 ODI points is considered clinically relevant. We will consider the sample size and power according to the MCID of ODI.

A minimum of 10 patients with chronic LBP are needed to produce stable estimates for each predicting variable. We will collect data on e.g. 20 potential predictive factors and screen these by logistic regression. If assuming we select four of these predictors for regression modelling, looking for an independent effect, then at least 40 patients with chronic LBP will be needed, 10 patients for each predictor.

We considered a series of scenarios for sample size calculations which resulted in an array of sample sizes between 200 and 800 patients necessary to ensure 80% power at a two-sided p of 0.05. If 5% of patients with acute LBP develop chronic LBP, 800 patients will have to be analysed. However, if 10% or 20% develop chronic LBP at 6 months follow-up only 400 and 200 patients respectively will be needed. Trends from our pilot study including 20 patients in Otago district, NZ show that an even smaller sample size might be sufficient – due to a rate of chronic patients of up to 30% in the pilot sample.

### Feasibility of the study

#### Patient recruitment

Patients in NZ will be recruited consecutively and prospectively in collaboration between the Department of Orthopaedic Surgery at Dunedin Hospital, UoO, and Best Practice Advocacy Centre Incorporated (BPAC Inc), Dunedin. GPs cooperating with BPAC Inc will ask patients who meet the study eligibility criteria to give their consent to be contacted by the research group at the UoO to participate in our study. Patients who give their consent may be given a detailed information sheet by their GPs about the study including contact details of the study coordinator. On the same day, contact details (telephone number, postal and email address) of these patients will be electronically forwarded in an automated fashion from the GP clinics to the research group at the UoO.

Patient assessment at different time points in our study will be conducted by research nurses at the UoO. At baseline a short screening interview on the telephone will be performed following a structured questionnaire. This interview will check the eligibility for enrolment (see inclusion criteria, Table 1). When a patient is classified as eligible, a document stating their signed consent and a first set of questionnaires (T0) will be sent out by post on the same day. At follow-up times (T1-4) research nurses will collect these patient-derived questionnaires and enter them into the central database. If not returning the questionnaire a first reminder will be sent out after one and a second after two weeks.

To optimise the number of completed questionnaires patients will be able to choose between a $NZ10 fuel, supermarket or book voucher as compensation for their expenditure of time after return of each of the five questionnaires. This level of compensation will not encourage unwilling participation. GPs will be compensated with the same choice of voucher for every referred patient.

Patients in CH will be recruited consecutively and prospectively at the realHealth Centre, Nottwil. Patient assessment and follow-ups will be conducted in parallel to the study arm in NZ by a research group headed by NA.

### Data collection

**Figure 1:**
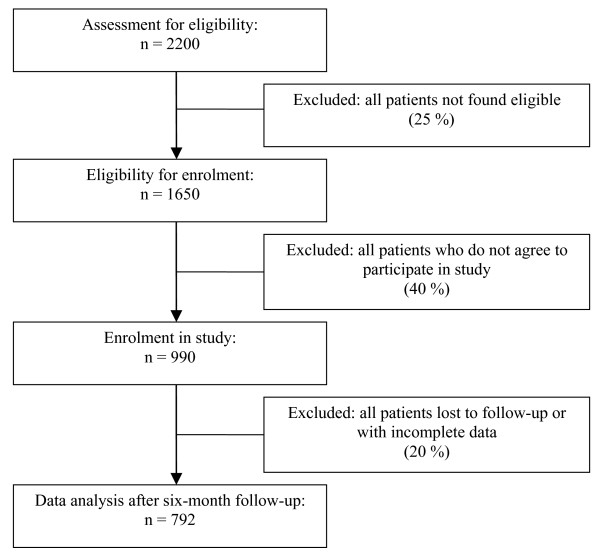
Recruitment and data collection.

2220 patients will be assessed for eligibility in NZ and CH respectively (Figure 1). It can be assumed that 75% of these screened patients will be found eligible for enrolment. Out of these 1650 patients it is estimated that 990 (60%) will agree to participate in this study. Up to 20% of the patients can be expected to be lost to follow-up, in the worst case scenario, so that an estimated 792 patients will be analysed.

The expected duration of the data collection phase of the study will be two years, comprising one and a half years of patient recruitment overlapping with two years of follow-up.

## Discussion

### Scientific significance

LBP is by far the most prevalent and most costly musculoskeletal problem in our society today [6,10,89,90]. Despite numerous previous studies, to our knowledge there has not been an attempt to comprehensively assess all potential factors which lead to the transition from acute to chronic LBP and the point in the disease progression when this occurs [7].

This study will assess a core set of outcome assessment tools for patients with acute LBP in a GP setting to assess which factors predict whether these patients are at risk of developing chronic LBP [91,92]. In addition, biomedical, psychological, social, and occupational baseline characteristics will be identified which may be found to influence the course from acute to chronic LBP. Furthermore, an appropriate time for follow-ups will be given to increase the likelihood of detecting this transition.

The international perspective of this study of patients in NZ and CH will be an asset in terms of generalizability and validation of our findings for use by others.

### Social and economic significance

The knowledge about the ideal assessment of predicting factors will help GPs to prevent patients from developing chronic LBP [90,93-98]. This might result in saving treatment and rehabilitation costs of chronic LBP for the benefit of health care providers and consumers [99].

## Abbreviations

ACC: Accident Compensation Corporation; Acute SF-12: acute version of short form 12 questionnaire; BPAC Inc: Best Practice Advocacy Centre Incorporated; BMI: Body Mass Index; CH: Switzerland; FABQ: Fear Avoidance Beliefs Questionnaire; GP: General practitioner; LBP: low back pain; MCID: Minimal clinically important difference; MMICS: Multinational Musculoskeletal Inception Cohort Study; MSPQ: Modified Somatic Perceptions Questionnaire; NZ: New Zealand; $NZ: New Zealand dollar; ODI: Oswestry Disability Index; PCS: Pain Catastrophizing Scale; RTW: return to work; UoO: University of Otago; VAS: Visual Analogue Scale; ZUNG: modified self-rating depression scale by Zung.

## Competing interests

NA is a member of realHealth International, Hitzkirch, Switzerland, providing assessment and treatment for patients with acute LBP in Switzerland by order and for account of health insurance companies. All other authors declare that they have no competing interests.

## Authors' contributions

MM is the principal investigator. He designed the study and is responsible for the protocol. NA is the team coordinator of the Swiss study arm. Together with MM he developed the key idea underlying this study. AE is MM's supervisor on psychology who structured the ideas about psychological, social, and work-related factors of LBP and significantly contributed to the selection of appropriate assessment instruments. The assessment of occupational factors is based on previous research by AE. CR, TZ, PaH, and SB contributed to the content of baseline and follow-up assessment. TB is the supervising spine surgeon of MM and contributed to the design of the biomedical assessment. PeH is responsible for the sample size and power calculation and for the design of the statistical analysis and the evaluation of the database. KS is responsible for the development of the documentation system and the data management. JCT is the project leader for NZ and overall supervisor of MM. All authors participated in the study design as well as read and approved the final manuscript.

## Pre-publication history

The pre-publication history for this paper can be accessed here:


